# Language selective or non-selective in bilingual lexical access? It depends on lexical tones!

**DOI:** 10.1371/journal.pone.0230412

**Published:** 2020-03-23

**Authors:** Xin Wang, Bronson Hui, Siyu Chen

**Affiliations:** 1 Department of Linguistics, Faculty of Human Sciences, Macquarie University, Sydney, Australia; 2 Department of Education, University of Oxford, Oxford, United Kingdom; 3 Second Language Studies Program, Michigan State University, East Lansing, Michigan, United States of America; 4 Department of Languages and Linguistics, University of Greenwich, London, United Kingdom; The Hong Kong Polytechnic University, HONG KONG

## Abstract

Much of the literature surrounding bilingual spoken word recognition is based on bilinguals of non-tonal languages. In the Mandarin spoken word recognition literature, lexical tones are often considered as equally important as segments in lexical processing. It is unclear whether and how lexical tones contribute to bilingual language processing. One recent study demonstrates that tonal bilinguals require the availability of both tonal and segmental information to induce cross-language lexical competition during bilingual lexical access, even without phonological overlap between the target and non-target language. The current study investigates whether overt phonological overlap between the target and non-target language would equally require both tonal and segmental information available to induce cross-language lexical competition. We employed two auditory lexical decision experiments with both Mandarin-English bilinguals and English monolinguals to test whether inter-lingual homophones (IH) would induce lexical competition from the non-target language, L1 Mandarin. Our results show that cross-language lexical competition was only observed with the presence of lexical tones, in addition to segmental overlap.

## Introduction

In the domain of spoken word recognition, recognizing an auditory stimulus is considered a process of matching spoken input with mental representations associated with word candidates, and then selecting the best candidate amongst those activated, each of which will be at least partially consistent with the input. Most theories of spoken word recognition (e.g., the Cohort model and the TRACE model) centre on the debate regarding how the sensory input activates lexical representations and how the best candidate is selected by eliminating alternatives [1–5]. Nevertheless, all current theories of spoken word recognition acknowledge the need to account for competition among candidates for lexical access and selection. These theories have been shaped by considerable evidence showing that words that reside in sparse phonological neighbourhoods (e.g., *wolf* has only a few phonologically similar words: *woof*, *wooly*, and *wool*.) are recognized more easily than words that reside in dense phonological neighbourhoods (e.g., *cat* has many phonologically similar words: *bat*, *at*, *cab*, *rat*, *chat*, *that*, *mat*, *cattle*, *pat*, *gnat*, and so on) [6–7]. This difference indicates that our lexical processor needs not only to interpret the unfolding sensory input, but also to inhibit the activation of non-target candidates. This inhibitory effect, which is required for successful spoken word recognition, has been extended to issues in bilingualism in order to understand how bilinguals recognize spoken words in one language that sound similar to words in the other [8–12]. In general, two main questions are being explored in this area. First, when bilingual listeners hear spoken words (especially interlingual homophones), do they activate word candidates in both languages or only those in the target language? Second, do bilingual listeners use language-specific phonetic features to select the word candidates in the target language?

The first issue has been widely discussed and debated in the bilingual literature, especially in the areas of bilingual visual word recognition [13] and bilingual language production [14]. In general, the debate has centered on two opposing views: a language selective view which predicts that linguistic input in one language should only activate the target language; and a language non-selective view which predicts that linguistic input in one language can induce co-activation of both languages. As a result, a language selective view would imply separate lexicons for two languages, while a language non-selective view suggests that the bilingual lexicon is integrated [15]. In the domain of visual word recognition, there is ample evidence showing that bilingual lexical access is language non-selective for recognizing inter-lingual homographs or cognates [16–20]. That is, visual input in one language can activate a bilingual’s other language, evidenced by faster or slower responses to inter-lingual homographs or cognates, depending on the task. In particular, cross-language masked priming studies have presented a strong test of language non-selectivity in bilingual lexical access for both within-script and cross-script readers who effectively processed prime-target pairs in different languages even being unaware of the existence of the prime words [21–28].

### Bilingual spoken word recognition

In the auditory domain, however, both empirical and modeling efforts to understand bilingual word recognition are more limited. It is less clear whether language non-selectivity equally applies in the auditory domain during bilingual lexical access, in particular, due to the rich sub-lexical cues encoded in auditory input. In fact, there are good reasons to test this, because visual input differs from auditory input in the following ways [10–11]. First, many languages, such as English and Spanish, use the same writing system such that the visual input, especially cognates or inter-lingual homographs, does not differentiate language membership, while the auditory input contains acoustic-phonetic cues that differentiate language membership (e.g., Spanish and English differ from each other in voice onset time). Second, the time course of stimulus presentation is different across the visual and auditory modalities: visual letters of a written word are usually presented simultaneously, while spoken words unfold over time. In fact, some evidence suggests that bilinguals might adopt language-specific processing strategies in speech perception, on the basis of language-specific acoustic-phonetic cues [29–30]. With this in mind, it is reasonable to posit that bilinguals might use language-specific cues to guide lexical access.

One line of research has adopted the visual world paradigm to investigate whether phonologically similar words across languages can activate both languages even under a monolingual experimental situation [9], [12], [31]. In this experimental procedure, bilingual participants are instructed in either L1 or L2 to move or click on target objects in a visual array and their eye fixations to objects within the array are recorded while they perform this task. Typically, each display consists of 4 different objects: the target object (e.g. *the speaker*); the cross-language competitor whose name in the non-target language is phonologically similar to the target (e.g., ‘the match’ in Russian, pronounced as /spi:tʃki/); and two filler objects. For example, Spivey and Marian [31] reported strong cross-language competition effects using this paradigm, reflected by a larger proportion of fixations to the cross-language competitor objects than to the phonologically unrelated distractors. That is, bilingual participants looked at the competitors for longer (52% vs. 37% of the time) and/or more often (31% vs. 13%) than the phonologically unrelated distractors. These differences were interpreted as the result of the spoken input activating both the target and the cross-language competitor. In turn, this was taken as support of the language non-selective view.

However, other researchers have stressed the importance of other factors that might constrain language selectivity or non-selectivity in auditory word processing. First, Weber and Cutler [12] demonstrated that language selectivity or non-selectivity in bilingual word recognition might depend on *language status* (i.e. whether the target language is L1 or L2). Adopting the same eye-tracking technique, they found that Dutch-English bilinguals fixated longer to competitor objects only when the target language was L2 English (e.g., the English target *kitten* activated the Dutch competitor *kist)*, but not when the target language was L1 Dutch (e.g., the Dutch target *kist* failed to activate the English competitor *kitten)*. This asymmetry suggests that co-activation in bilingual word recognition is not unconditional, but rather depends on the characteristics of the auditory input. Second, using the same experimental paradigm, Ju and Luce [9] found that Spanish-English bilinguals fixated cross-language competitors (i.e., the non-target object whose English name *pliers* is phonologically similar to the Spanish target *playa* 'beach') more frequently than phonologically unrelated distractors (e.g., *ojo* 'eye'), but only under the condition where the Spanish target words were altered to have an English appropriate voice onset time (VOT). These results suggest that bilingual listeners might be sensitive to language-specific cues so as to guide lexical access.

Other experimental paradigms have also been used to investigate the issue of language selectivity or non-selectivity in the auditory domain. For instance, in a cross-modal priming paradigm, Dutch-English bilinguals completed a lexical decision task on visual word targets that were preceded by auditory word primes [11]. The critical conditions for comparison were: (1) targets were inter-lingual homophones of primes (IHs, e.g. *lease* primed by /liːs/, where *lease* sounds like *lies* ‘groin’ /liːs/ in Dutch); and (2) non-IH prime-target pairs (e.g. *frame* primed by /freɪm/, which is not a lexical item in Dutch). The authors found that reaction times for the IH condition were significantly slower than those for the non-IH targets. Given that longer reaction times reflect a larger cohort [32], the results showed that the cohort size formed upon hearing the IH (e.g., /liːs/) was larger than that formed upon hearing the control prime (e.g, /freɪm/), suggesting that both English and Dutch meanings of the inter-lingual homophones were activated. The inhibitory effect generated on the IH prime-target pairs was interpreted as cross-language lexical competition, as a result of language non-selectivity.

Similar effects for IHs were also reported by Lagrou, Hartsuiker, and Duyck [10] in a more straightforward way with Dutch-English bilinguals who performed an auditory lexical decision task in both L1-Dutch and L2-English. For example, in their experiments, Dutch-English bilinguals responded to IHs in Dutch (e.g, *bij*, pronounced similarly to ‘*bye*’ in English) more slowly than non-IHs in Dutch (e.g., *vol)*. However, monolingual Dutch listeners did not show this inhibitory effect on IH words. Along the same line, they tested the same bilinguals with English IH and non-IH words in an auditory lexical decision task and obtained the same pattern, compared to monolingual English listeners. These results were taken as evidence to support language non-selective access in bilingual language processing because the inhibitory effect was driven by the cross-language lexical competition.

Furthermore, both Shulpen et al. [11] and Lagrou et al. [33] investigated whether sub-lexical cues (i.e. language-specific acoustic information, like accent) would modulate cross-language activation so as to guide lexical access. They found bilinguals were sensitive to language-specific sub-phonemic cues, but lexical access was still language non-selective. That is, both languages were active and competing for access in processing, regardless of accent. Interestingly, the same authors reported that bilingual auditory word recognition in a sentence context could be modulated by semantic constraints and speaker accent, which did not restrict cross-language activation either [33]. These results suggest that language-specific speech cues were exploited by bilingual listeners to guide lexical access.

Thus, an overview of the relevant literature seems to suggest that bilingual lexical access in the auditory modality is largely language non-selective, but is also sensitive to language-specific phonetic cues that can be used to guide lexical access. In particular, an interesting question to ask is the extent to which bilingual listeners use the language-specific speech cues in cross-language lexical competition and access. To date, the majority of research has primarily focused on segmental information (e.g., phonemes) in triggering language co-activation (i.e. cross-language lexical activation/competition); it remains underexplored whether and to what extent supra-segmental information (e.g., lexical tones) would affect word recognition in bilinguals of tonal languages. Thus, the primary objective of the present study is to investigate the role of lexical tone representation in cross-language processing in tonal bilinguals (e.g., Mandarin-English bilinguals).

### Lexical tone processing in monolinguals and bilinguals

Belonging to the Sino-Tibetan language family, Mandarin Chinese is a tonal monosyllabic language, which utilizes four different tones to disambiguate lexical meanings [34]. For example, the Mandarin word *ma* can refer to “mother” when pronounced in Tone 1 (high flat), “hemp” when pronounced in Tone 2 (rising), “horse” when pronounced in Tone 3 (low dipping) and “scold” when pronounced in Tone 4 (falling). Thus, lexical access in Mandarin needs to involve both segmental information (i.e., consonants and vowels) and supra-segmental information (i.e., lexical tones). By contrast, in English, pitch contours do not alter lexical meanings. In addition, Mandarin syllables predominantly follow a CV (consonant-vowel) structure, with the only exceptions being syllables which include the nasals /n/ and /ŋ/ in the coda position [35]. Because a Mandarin syllable can be articulated in as many as four tones, most tonal syllables are homophones of other morphemes/words/characters. Given the limited number of legal syllables in Mandarin, one syllable is shared by eleven characters on average [36].

Because Mandarin tones are lexical, it has been reported in the literature that Mandarin tones are a critical cue in constraining spoken word recognition [37–41]. In an auditory priming paradigm, where participants performed lexical decision on auditory targets following auditory primes, Lee [37] investigated the role of lexical tones in word recognition. In this study, there was a total of four conditions differing in the phonological relationship between primes and targets: 1) prime-target overlap in only segmental information (e.g., *lou3* ‘hug’—*lou2* ‘hall’); 2) prime-target overlap in only tone (e.g., *cang2* ‘hide’—*lou2* ‘hall’); 3) prime-target overlap in both segments and tone (e.g., *lou2* ‘hall’—*lou2* ‘hall’); and 4) no prime-target overlap (e.g., *pan1* ‘climb’—*lou2* ‘hall’). Under two different ISIs (prime-target inter-stimulus interval: 250ms and 50ms), reliable priming was only observed in the condition where both tones and segments overlapped between primes and targets. The author concluded that segmental overlap alone was not sufficient for facilitation on target recognition, and thus lexical tones were critical in producing priming.

Further, Malins and Joanisse [38] used the visual world paradigm to compare the role of tonal information and segmental information in Mandarin spoken word recognition. Native Mandarin speakers were presented with auditory Mandarin words and instructed to press a button on a keypad corresponding to the position of a target picture in a visual array. The visual stimulus display consisted of the target (e.g., *chuang2* ‘bed’), a competitor item whose name overlapped phonologically with the target in either segments (e.g., *chuang1* ‘window’), onset and tone (e.g., *chuan2*, ‘ship’), rhyme and tone (e.g., *huang2* ‘yellow’), or only tone (e.g., *niu2* ‘cow’), in addition to two unrelated distractors. The authors found that the time course over which listeners resolved competition between items differing in segments was comparable to that over which listeners resolved competition between items differing in tone. Following this, they concluded that tones not only constrained the cohort size, but also played a role that was comparable to segmental information in Mandarin spoken word recognition.

Therefore, it is clear that lexical tones provide important independent cues for lexical access within a tonal language, yet there is limited evidence showing how lexical tones play a role in cross-language processing. In fact, research has primarily explored how tonal bilinguals process a non-tonal language differently at the perceptual level due to their extensive experience with lexical tones [42,43]. For instance, Ortega-Llebaria et al. [43] demonstrated that Mandarin speakers of English were more sensitive to F0 than Spanish speakers of English and native English speakers when recognizing English words; in addition, Mandarin speakers were faster retrieving a falling F0 than a rising F0 in an English lexical decision task. This study suggests that tonal bilinguals process pitch contour differently even in a non-tonal language, compared to non-tonal bilinguals and monolinguals. However, this evidence does not offer insights of how tonal information would affect word recognition in the non-tonal language.

One recent study tapping into the effect of lexical tones in cross-language processing at the lexical level employs a translation task with Mandarin-English bilinguals [44]. Tonal bilinguals were instructed to select the correct Mandarin translations, which were visually presented on the computer screen, of the auditory English words. The auditory English words were manipulated in pitch contour to either match with the tones of the Mandarin translations or mismatch with the tones of the Mandarin translations. Tonal bilinguals were found to be sensitive to the manipulation of pitch contours in English words. While one can argue that the tonal effect in the translation task could also be driven by the visually presented Chinese characters, another recent study demonstrated the tonal effect in cross-language processing in a more straightforward but implicit way. Wang, Wang, and Malins [45] demonstrated that Mandarin-English bilinguals implicitly accessed their L1 Mandarin words when recognizing auditory English words in the visual world paradigm and that this cross-language lexical activation/competition was sensitive to lexical tones. For example, in the visual world paradigm, when Mandarin-English bilinguals listened to the word ‘*rain’*, whose translation in Mandarin is *yu3*, they were instructed to pick the target picture of *rain* among an array of 4 pictures on the computer screen. Among the 4 pictures, there was a competitor whose name in Mandarin was either a homophone of the Mandarin translation of the target (e.g., ‘*feather*’–*yu3*) or an item that only overlapped with the Mandarin translation in segments but not in tones (e.g., ‘fish’—*yu2*). The eye-movement data only showed significance in the homophone condition where both segments and tones overlapped with the Mandarin translations of the targets, suggesting that participants landed their eye fixations on the competitors (e.g., *‘feather’*) before picking the target (e.g., *‘rain’*). This competition effect was interpreted as evidence of lexical competition corresponding to the competitor picture and that the source of the lexical activation in Mandarin was due to the phonological overlap between the competitor and the target translation in both segments and tones. This evidence indicated that Mandarin-English bilinguals implicitly accessed their L1 Mandarin when recognizing L2 words and that lexical activation in L1 Mandarin was driven by both segments and tones. This study was the first to demonstrate a cross-language tonal effect in English only, a non-tonal language, without any manipulation on pitch contour or phonological overlap between the target and non-target language in the input.

Thus, it remains unclear whether a pitch contour on an English word would have any effect in cross-language lexical activation in Mandarin in a direct and explicit way without involving the translation process. One way to address this issue is to see whether words pronounced similarly/the same across languages would be able to elicit language co-activation with or without tones, like in [11] and Lagrou et al. [10] [33] where cross-language homophones elicited lexical competition with non-tonal bilinguals. Homophones are words that have the same pronunciation but differ in meaning, spelling, or grammatical class [46]. In the same vein, **inter-lingual homophones (IH)** are words that are pronounced similarly across languages but differ in meaning, spelling, or grammatical class. Thus, IHs share segments but not tones for Mandarin-English bilinguals. The main purpose of the current study is to investigate whether lexical tones are crucial in cross-language lexical activation/competition when tonal bilinguals are exclusively processing a non-tonal language. Given the compelling evidence within and across languages that supra-segmental information is crucial in activating Mandarin words, we hypothesize that Mandarin-English inter-lingual homophones sharing *only* segments are not sufficient to trigger parallel language activation as in non-tonal bilinguals and that lexical tonal information needs to be available along with segments.

### The present study

We aim to test whether the presence of lexical tones is critical in cross-language lexical competition, thus, tonal manipulation can be achieved with English target words in order to measure the lexical activation in the non-target tonal language (e.g., Mandarin). Experiments 1–2 were designed to compare results from Mandarin-English bilinguals in order to test whether IHs with versus without lexical tones would produce similar cross-language inhibitory effects as in Lagrou et al. [10]. We instructed bilingual participants to identify words in their L2 English and presented inter-lingual homophones either as naturally produced native English words (Experiment 1) or English words superimposed with lexical tones (Experiment 2). We hypothesize that lexical tones are obligatory in guiding lexical access to L1 Mandarin; thus, IHs with lexical tones will induce cross-language lexical competition, in contrast to no cross-language lexical competition in IHs without lexical tones. In order to confirm that this difference is due to bilinguals’ knowledge of Mandarin, we also tested English native speakers who should not show any difference in this manipulation of pitch contour. In other words, monolingual English listeners should not show any difference regardless of whether IHs are natural or superimposed with lexical tones as they do not have any knowledge of Mandarin. *Note that our logic is not to compare Mandarin-English bilinguals and English monolinguals within Exp 1 or 2*, *but to compare within bilinguals and monolinguals to show that the presence of lexical tones produced cross-language lexical competition effect in bilinguals but not monolinguals*.

## Experiment 1 (Auditory lexical decision task)

### Materials and methods

#### Participants

Twenty-three Mandarin-English bilinguals and 22 English monolinguals from the University of Oxford participated in Experiment 1 for a monetary compensation. All the participants provided their written consent forms for the study which was approved by the Departmental Research Ethics Committee (DREC) in accordance with the procedures prescribed by Oxford University for ethical approval of all research involving humans (CUREC). The bilingual participants were asked to report their English proficiency with respect to the four skills (i.e., speaking, listening, writing and reading), using a Likert scale from 1 (very poor) to 7 (native-like), as well as their IELTS scores to be admitted to study in the UK. Means and SD are reported in Table 1. The bilingual participants were not informed that their L1 Mandarin knowledge would be relevant to the experiment. The whole experiment was conducted in English.

**Table 1:** Means (SD) based on bilingual participants’ self-ratings of their English language skills on a 1–7 Likert scale (1 = very poor and 7 = native like) and IELTS scores in Experiments 1 and 2.

	IELTS	Reading	Writing	Speaking	Listening
Experiment 1 *(n = 22)*	7.16 (0.3)	5.9 (0.5)	5.0 (0.9)	5.5 (0.8)	5.8 (0.7)
Experiment 2 *(n = 21)*	7.14 (0.4)	5.8 (0.5)	4.7 (0.8)	5.2 (0.5)	5.5 (0.7)
*t(df)*	0.16 (41)	0.66 (41)	1.15 (41)	1.47 (41)	0.94 (41)

#### Stimuli

Three types of stimuli–interlingual homophones (IHs), non-interlingual homophones (non-IHs) and non-words–were selected for the study. In selecting interlingual homophones, a systematic comparison of Mandarin and English phonemes resulted in 24 pairs of phonemes that were considered to be sufficiently similar across Mandarin and English, including 10 vowels and 14 consonants [47]. There could, in theory, be 140 CV syllables in Mandarin sounding similar to English syllables. We, highly proficient Mandarin-English bilinguals, compiled a table to see how many possible CV syllables are permissible and sound similar across Mandarin and English (See S1 Appendix). Among these, 50 are legal in both languages, 50 are legal only in Mandarin, 17 are legal only in English, and 23 are illegal in both languages. In Mandarin, each syllable can be articulated with different lexical tones; thus, taken together, 150 meaningful tonal syllables in Mandarin correspond to 50 meaningful syllables in English (e.g., both *法* (law), pronounced as /fa/ in Tone 3, and *发* (hair), pronounced as /fa/ in Tone 4, correspond to *far* in English). In addition, these Mandarin syllables (words) are semantically unrelated to their counterparts in English.

These 50 IH items were then rated by 5 highly proficient Mandarin-English bilinguals on a Likert scale from 1 (completely different) to 7 (the same) on their similarity in pronunciation between Mandarin and English. All the raters were native speakers of Mandarin and had received undergraduate and postgraduate training in General Linguistics or English Linguistics, such that they had some training in understanding the linguistic similarity between two languages. They were given English monosyllabic words (e.g., *bay*) and their counterparts in Mandarin (e.g., 被 /bei4/ ‘quilt’) and assessed how similar they were after reading aloud each pair in both languages. In the similarity rating, the most frequent Chinese character (word) among characters of the same segments/syllables but of different tones, based on the SUBTLEX-CH database [48], was chosen to represent the Mandarin counterparts. Eventually, we selected 37 interlingual homophones (e.g. *me*—/mi4/ as in 密 ‘secret’), having a cut-off mean score of 4.0 or above in the ratings across the 5 bilingual raters (see S2 Appendix). Another 37 monosyllabic non-IHs (e.g., *sale*) were selected and matched item by item in their frequency and phonological neighborhood density with the IHs, based on the Irvine Phonotactic Online Dictionary (IPhOD) database [49] (See Table 2 & S3 Appendix). An independent *t*-test confirmed that there was no statistically significant difference in log frequency (*t* (72) = .05, p = .96) between the IHs (M = 3.12, SE = 2.56) and the non-IHs (M = 3.11, SE = 2.57). There was also no statistical difference in the phonological neighborhood density (*t* (72) = .99, p = .32) between the IHs (M = 39.49, SE = 1.18) and the non-IHs (M = 39.65, SE = 4.42). Controlling the number of phonemes was unrealistic because there are not sufficient English words of CV (Consonant-Vowel) structures to be selected as non-IHs, when both frequency and phonological neighborhood density were matched. This limitation is due to the fact that most Mandarin syllables do not have a coda while English monosyllabic words with a CV structure are less common [35]. As a result, the IHs consist of 8 items of 3 phonemes and 29 items of 2 phonemes; by contrast, the non-IHs all contain 3 phonemes. Nasal codas, like /n/, are considered independent phonemes, not part of the vowel in the CV syllables. In addition, 74 monosyllabic non-words were generated from the same database in [49], all of which contain 3 phonemes.

**Table 2:** Stimulus characteristics of Experiments 1–2: Means (SD) of log frequency, phonological neighborhood density (PND), and number of phonemes.

	Frequency	PND	Number of phonemes
**IH**	3.12 (3.34)	39.49 (7.21)	2.25 (0.44)
**Non-IH**	3.11 (3.36)	39.65 (8.64)	3 (0)
*t*(df)	-.05 (72)	.99 (72)	10.58 (72) **

#### Auditory recording

The speaker recording the stimuli was a 25-year-old highly proficient female simultaneous Mandarin-English bilingual. She grew up in a household where her father was a native speaker of English and her mother was a native speaker of Mandarin. As a result, she reported acquiring both languages simultaneously. She reported speaking mostly Mandarin at home and mostly English at school. To ensure that the selected bilingual could produce native English words, we asked her to read aloud an English passage and her voice was recorded. Six native English speakers were asked to judge the passage on a 1–5 Likert scale (1 = native English speaker, no accent; 5 = strong foreign accent) about the native-ness of the Mandarin-English bilingual’s English. Out of the six raters, five rated her English as 1, and one rated 2. All stimuli were recorded using the open source software Audacity, version 2.0.3 [50] at 44.1 kHz with a recorder in a quiet room. Prior to the actual recording, the speaker was given time to familiarize herself with the stimuli and read aloud for practice. All tokens were trimmed for programming purposes and normalized to -1.0 dB for amplitude.

#### Procedure

Participants were tested in a quiet room and were wearing a headset in front of a testing computer. Prior to the experiment, they were given a written instruction of the task in English. Each trial started with a 500ms fixation (+) on the center of the screen, using a black font size of 30 against a white background, followed by the presentation of the auditory stimulus through the headset. Immediate to that, the participants were expected to press either the YES or NO button. They were instructed to press YES if the auditory stimulus was a word in English; otherwise, press NO. Visual feedback, either ‘Correct!’ or ‘Incorrect’ as appropriate, was presented at the bottom of the screen for 200ms immediately after the response. The between trial interval was 250ms. Responses were recorded, and reaction times (RTs) were recorded from word onset until the motor response on the YES or NO key on the keyboard. All the trials were programmed for the presentation of stimuli in a random order by E-Prime 2.0 [51].

## Results and discussion

Participants who made errors on more than 30% of the total trials were excluded from the analysis. As a result, 22 out of 23 bilingual participants and 22 monolingual participants were included in the final analysis. Statistical analyses were performed using linear mixed-effects models [52,53]. Unlike more traditional ANOVAs, mixed-effects models take raw unaveraged data as input and incorporate both random effects of participants and items within a single analysis. In addition, we employed maximal random-effect structures in the models and included random slopes for factors of repeated measures [54], to avoid Type I errors. The fixed-effect factors were Word Type (IHs vs. non-IHs) and Group (bilinguals vs. monolinguals). Subjects and items were random factors. The lmerTest functions from the lme4 package (version 1.1–7) in R were used (version 3.1.0; CRAN project; The R Foundation for Statistical Computing, 2008). In error analysis, the binomial function (i.e., glmer) was employed to report the statistical significance of error rates across conditions; in reaction time analysis, the lmer function was employed to show the statistical significance of response times across conditions. Following standard conventions, any t-value greater than 2.0 or p-value smaller than .05 was deemed significant.

Because non-words were not of our theoretical interests in lexical decision, we only presented and analyzed data that reflect our factorial design: Group (monolingual vs. bilingual) x Word Type (IH vs. non-IH). Mean error rates and response times for IH and non-IH words are presented in Table 3. The overall analysis of error rates showed neither main effect of Group *(z =* .*38*, *p =* .*71)*, nor main effect of Word Type (*z = 1*.*72*, *p =* .*09*). There was a marginal interaction between Group and Word Type (*z = 1*.*85*, *p =* .*01 <* .*06*). Restricting the analysis to each group, the error rates of the bilingual results showed no statistical difference between the non-IH and IH conditions (*z = 1*.*6*, *p =* .*11)*. Similarly, the error analysis of the monolingual results showed no significant difference between the non-IH and IH conditions *(z =* .*04*, *p =* .*97)*. These results indicate that neither group found any type of words particularly difficult to process; however, the non-IH words were slightly more difficult (compared to the IH words) for bilinguals than monolinguals (i.e., interaction).

**Table 3:** Mean offset reaction times (RTs in milliseconds) (SD) and error rates (ERs in percentages) (SD) (Experiment 1 & 2).

			**IH**	**Non-IH**	
**Error Rates**	**Exp 1**	*Bi (n = 22)*	13.4 (.34)	19.1 (.39)	
	(no tones)	*Mono (n = 22)*	12.4 (.33)	12 (.33)	
	**Exp 2**	*Bi (n = 21)*	23.5 (.42)	16.1 (.37)	
	(with tones)	*Mono (n = 20)*	29.9 (.46)	16.2 (.37)	
			**IH**	**Non-IH**	**Difference** (IH vs. non-IH)
**Reaction Times**	**Exp 1**	*Bi (n = 22)*	622 (310)	633 (340)	-11
	(no tones)	*Mono (n = 22)*	540 (254)	475 (233)	65*
	**Exp 2**	*Bi (n = 21)*	847 (425)	722 (385)	125***
	(with tones)	*Mono (n = 20)*	604 (349)	509 (303)	95**

Analyses on reaction times were based on responses to the word offsets. The choice of using the word offset measures was due to the variation in the duration of the critical word stimuli (Min. = 346ms, Max. = 981ms, Mean = 617ms, SD = 133ms). The average word durations for IHs, non-IHs and nonwords are: 555ms, 681ms, and 930ms. Therefore, the offset measure was believed to be more sensitive than the onset measure, free from the confounding variation of the stimuli durations [55]. The offset measures were calculated as the differences between the latencies logged by E-Prime (i.e., from the stimuli onset to the motor response on the keyboard) and the duration of the stimuli as measured using Praat [56]. In analyzing the data, reaction times outside 2SD above or below the mean were excluded from analysis (3%), as were trials on which an error occurred (16.1%).

The overall maximal mixed-effects analysis of the RTs showed a main effect of Group *(t = 2*.*71 p <* .*01)* and an interaction between Group and Word Type (*t = 2*.*87*, *p <* .*01*). However, there was no main effects of Word Type (*t = 0*.*097*, *p =* .*93*). These results show that the bilingual participants responded to L2 English spoken targets much more slowly than their monolingual counterparts and these two groups responded to the experimental manipulation (IH vs. non-IH) differently. Restricting the analysis to just the bilingual group, the mixed-effects analysis of the RTs showed no main effect of Word Type (*t =* .*18*, *p =* .*86*). Thus, bilinguals treat both IHs and non-IHs similarly, without demonstrating the evidence of activating the Mandarin lexicon so as to interfere lexical access in English. However, when restricting the analysis to just the monolingual group, the mixed-effects analysis of the RTs showed a main effect of Word Type (*t = 2*.*29*, *p =* .*025 <* .*05*). This result indicates that monolinguals responded to IHs more slowly than non-IHs. The outcome of the statistical models analyzing both groups is presented in Table 4.

**Table 4:** Linear mixed-effects analysis results for Experiment 1.

Monolinguals and Bilinguals						
*Random effects*:						
Groups	Name	Variance	Std.Dev.		Corr	
itemN	(Intercept)	11016	104.96			
	groupmono	1150	33.91		-0.66	
subject	(Intercept)	6450	80.31			
	wtypenon-IH	1218	34.90		0.21	
Residual		39136	197.83			
*Fixed effects*						
	Estimate	Std. Error	df	t value		Pr(>|t|)
(Intercept)	600.384	25.530	87.214	23.517		<2e-16 ***
wtypenon-IH	2.729	28.101	72.667	0.097		0.9229
groupmono	-73.293	27.096	43.764	-2.705		0.0097 **
wtypenon-IH:groupmono	-58.530	20.401	37.831	-2.869		0.0067 **
Bilingual Group						
*Random effects*:						
Groups	Name	Variance	Std.Dev.	Corr		
itemN	(Intercept)	10463	102.29			
subject	(Intercept)	8539	92.41			
	wtypenon-IH	1790	42.31	0.11		
Residual		46067	214.63			
*Fixed effects*:						
	Estimate	Std. Error	df	t value		Pr(>|t|)
(Intercept)	598.424	27.269	45.734	21.945		<2e-16 ***
wtypenon-IH	5.022	28.494	62.241	0.176		0.861
Monolingual Group						
*Random effects*:						
Groups	Name	Variance	Std.Dev.	Corr		
itemN	(Intercept)	7738.6	87.97			
subject	(Intercept)	4548.1	67.44			
	wtypenon-IH	598.4	24.46	0.39		
Residual		32840.1	181.22			
*Fixed effects*:						
	Estimate	Std. Error	df	t value		Pr(>|t|)
(Intercept)	526.18	21.56	53.60	24.405		<2e-16 ***
wtypenon-IH	-53.72	23.42	64.97	-2.293		0.0251 *

p < 0.1.

* p < 0.05.

** p < 0.01.

*** p < 0.001.

It was predicted that if bilingual lexical access is language non-selective, the bilinguals would respond more slowly to the IHs than to the non-IHs (i.e. showing IH inhibitory effects due to cross-language lexical competition). On the other hand, if lexical access is language selective, IH effects would not be observed. The current results show a clear pattern in favor of language selectivity. The critical result is that bilinguals did not show any disadvantage in responding to IHs, which is contradictory to previous results in [10,11], [33] and indicates that lexical access is language-specific in the current experiment. In addition, bilinguals and monolinguals showed a contrast in responding to IHs vs. Non-IHs in the above analysis, as monolinguals showed an advantage in processing the non-IHs over the IH items. It is safe to rule out that the inhibitory effect on the IHs was due to cross-language lexical competition because the monolinguals did not have any knowledge of Mandarin. We believe that this difference showed in monolinguals is due to the characteristics of our stimuli in the IH and non-IH conditions; because most of the IHs are open syllables to match with their Mandarin counterparts while most of the non-IHs are closed syllables with other matched psycholinguistic variables. Due to the constraints in selecting appropriate stimuli in both languages to meet certain criteria, the difference of the phonotactic structures between Mandarin and English was impossible to avoid because English has quite a small number of open monosyllabic words and Mandarin is a prevalently open-syllabic language. On the other hand, this contrast indicates that bilinguals process their L2 English differently from monolinguals; otherwise, they should also have showed inhibitory effects on the IHs. We will return to this later in General Discussion, with regard to whether/how this inhibitory effect observed in monolinguals would affect our interpretation of the results.

Thus, Experiment 1 did not show cross-language lexical competition effects with inter-lingual homophones when lexical tones were absent. To further investigate this, Experiment 2 was designed to understand whether the same stimuli would produce cross-language lexical competition with the presence of lexical tones. In order to test this hypothesis, we superimposed Mandarin tones onto the English words and non-words in Experiment 2.

## Experiment 2 (Toned auditory lexical decision task)

### Materials and methods

#### Participants

Participants were recruited from the same population as those in Experiment 1, consisting of 22 Mandarin-English bilinguals and 21 English monolinguals. The self-reported English proficiency with respect to the four skills (i.e., speaking, listening, writing and reading) and IELTS scores are reported in Table 1. Two sample independent t-tests showed that the reported proficiency and the IELTS scores in this sample was not statistically different from that in Experiment 1 (Table 1).

#### Stimuli

The materials and design of Experiment 2 were the same as Experiment 1, except that Mandarin tones were superimposed onto all stimuli. For the IHs, the tone chosen for superimposition was that of the most frequent Chinese character corresponding to the English IH in pronunciation, based on the SUBTLEX-CH database [48]. For example, the IH *my* or /maɪ/ corresponds to at least nine Chinese characters, the most frequent of which is 买 ‘to buy’ /maɪ3/ (Pinyin: mai3). Thus, the third tone was chosen for the superimposition of *my*. As the non-IH was selected to match the IH item-by-item in both frequency and phonological neighbourhood density, we superimposed the same tone used for its matched IH. The four tones in Mandarin were assigned randomly for nonwords.

#### Auditory recording

To test whether native speakers of Mandarin are sensitive to tonal information when processing English, we superimposed Mandarin tones of the IHs onto English in Experiment 2. That is, each homophone was superimposed with a Mandarin tone that corresponds to the tone of its inter-lingual counterpart. Tokens were recorded by the same simultaneous bilingual speaker as in Experiment 1. To create the experimental tokens, we followed the same procedure of tone superimposition adopted in [44], and used natural speech rather than synthesized speech as it was reported that synthesized speech imposed more difficulty for bilingual listeners [57]. The implementation of tone superimposition was conducted as follows. A native Mandarin speaker (the experimenter) trained the Mandarin-English bilingual to produce 4 Mandarin tones on a given syllable to ensure that the speaker could naturally produce Mandarin tones. To produce Mandarin tones with novel syllables in English, it is easier and more consistent for the speaker to produce four different tones in the Tone 1 –Tone 4 sequence for each syllable. After some training and practice, the Mandarin-English bilingual was comfortable and proficient in producing English words with 4 different Mandarin tones. In addition, prior to the recording session, a sequence of the four Mandarin tones for a novel syllable /p^h^a/ [Pinyin: pa], (i.e., pa1, pa2, pa3 and pa4), was played to the speaker as an example to follow. Thus, the bilingual speaker pronounced each given English word and non-word, and then pronounced each word/non-word with 4 Mandarin tones in the sequence from Tone 1 to Tone 4. Throughout the entire recording session, no Mandarin was used at all. Furthermore, recordings were independently judged by another native speaker of Mandarin so that tonal tokens evaluated as ‘awkward’ were re-recorded. These re-recorded items were all nonwords. Only toned syllables matched with experimental items/designs were used in testing. All the auditory stimuli were trimmed for programming purposes and normalized to ensure all the tokens have the same amplitude (i.e., -1.0dB).

#### Procedure

The procedure of Experiment 2 was the same as Experiment 1.

## Results and discussion

The data trimming and analysis procedure were the same as in Experiment 1. As a result, 1 bilingual participant and 1 monolingual participant were excluded from the analyses because their error rates were larger than 30%. Similarly, along with the results from Experiment 1, mean response times and error rates for IHs and non-IHs are presented in Table 3. The error rate analysis of the bilingual results showed no statistical difference between the IH and non-IH conditions (*z = 1*.*67*, *p =* .*094)*. However, the monolingual results showed significant difference between the IH and non-IH conditions (*z = 3*.*20*, *p <* .*01)*. This means that monolinguals found the IH words more difficult to process than the non-IH words, while this was not the case for bilinguals. A combined analysis of both groups in error rate showed a marginal main effect of Group (*z = 1*.*89*, *p =* .*059)*. However, there was neither main effect of Word Type *(z = 1*.*64*, *p =* .*10)* nor interaction between Word Type and Group (*z = 1*.*58*, *p =* .*11)*. These results indicate that bilinguals encountered more difficulty during lexical processing in their L2 than monolinguals.

Same to Experiment 1, the analysis on reaction times was based on word offsets to responses. Reaction times above or below 2SD from the mean were excluded in analysis (2.2%), as were trials on which an error occurred (21.6%). The overall maximal mixed-effects analysis of the RTs showed the main effects of Word Type (*t = 2*.*57*, *p =* .*01 < 0*.*05*) and Group (*t = 3*.*58*, *p < 0*.*001*). There was no interaction between Word Type and Group (*t =* .*22*, *p =* .*82*). Similar to Experiment 1, these results showed that the bilingual participants responded to L2 English spoken targets much more slowly than their monolingual counterparts. However, different from Experiment 1, the IH items became more difficult for bilinguals with the presence of lexical tones, compared to the non-IH items (125ms difference). Restricting the analysis to just the bilingual group, the mixed-effects analysis of the RTs showed a main effect of Word Type (*t = 2*.*47*, *p =* .*017 < 0*.*05*). Unlike the results from Experiment 1, with the presence of lexical tones, the bilinguals treated IHs and non-IHs differently by responding to the IH words significantly more slowly than the non-IH words. This result suggests that the Mandarin lexicon was activated so as to create lexical competition in processing English words. These results are consistent with previous studies [10,11], [33]. In other words, lexical tones are a critical cue for lexical access in the non-target Mandarin when Mandarin-English listeners are processing English only. When restricting the analysis to just the monolingual group, the mixed-effects analysis of the RTs showed a main effect of Word Type (*t = 2*.*88*, *p <* .*01*). This result indicates that the monolinguals responded to the IHs much more slowly than the non-IHs, similar to the monolingual results in Experiment 1. The outcome of the statistical models is presented in Table 5.

**Table 5:** Linear mixed-effects analysis results for Experiment 2.

Monolinguals and Bilinguals						
*Random effects*:						
Groups	Name	Variance	Std.Dev.		Corr	
itemN	(Intercept)	20695.2	143.86			
	groupmono	6449.3	80.31		-0.38	
subject	(Intercept)	51774.9	227.54			
	wtypenon-IH	670.3	25.89		-0.12	
Residual		113970.9	337.60			
*Fixed effects*:						
	Estimate	Std. Error	df	t value		Pr(>|t|)
(Intercept)	908.961	57.137	54.561	15.908		< 2e-16 ***
wtypenon-IH	-102.654	39.968	63.020	-2.568		0.012598 *
groupmono	-269.734	75.445	40.968	-3.575		0.000914 ***
wtypenon-IH:groupmono	-7.925	35.590	37.767	-0.223		0.824981
Bilingual Group						
*Random effects*:						
Groups	Name	Variance	Std.Dev.	Corr		
itemN	(Intercept)	18526	136.11			
subject	(Intercept)	74223	272.44			
	wtypenon-IH	3619	60.16	-0.01		
Residual		149272	386.36			
*Fixed effects*:						
	Estimate	Std. Error	df	t value		Pr(>|t|)
(Intercept)	909.29	65.92	25.33	13.793		2.78e-13 ***
wtypenon-IH	-103.18	41.84	49.78	-2.466		0.0171 *
Monolingual Group						
*Random effects*:						
Groups	Name	Variance	Std.Dev.	Corr		
itemN	(Intercept)	20304.6	142.49			
subject	(Intercept)	29955.1	173.08			
	wtypenon-IH	187.4	13.69	-1.00		
Residual		76225.9	276.09			
*Fixed effects*:						
	Estimate	Std. Error	df	t value		Pr(>|t|)
(Intercept)	637.64	47.24	32.36	13.50		7.66e-15 ***
wtypenon-IH	-109.30	37.95	67.13	-2.88		0.00533 **

p < 0.1.

* p < 0.05.

** p < 0.01.

*** p < 0.001

### Combined analysis of Experiment 1 and 2

To confirm our hypothesis, it is crucial to demonstrate that superimposed tones indeed altered the responses to different types of words for bilinguals but not monolinguals. That is, lexical tones superimposed onto English words activated lexical representations in Mandarin to induce lexical competitions in IHs but not non-IHs for bilinguals; as a result, the bilinguals’ responses to the IH words were slowed down compared to the non-IH words in Experiment 2 in contrast to Experiment 1. In addition, this change, based on the knowledge of the non-target language (Mandarin), should not be observed in monolinguals. Thus, it is critical to show three-way interaction in our statistical model across experiments: Word Type x Group x Tone. Here, ‘Tone’ was a coded variable indicating that words/items in Experiment 1 were untoned and those in Experiment 2 were toned.

First, we ran linear mixed-effects analysis, with maximal random-effect structures, to understand whether the response differences between IHs and non-IHs with or without lexical tones differed between these bilinguals and monolinguals. That is, to confirm our hypothesis that the toned words elicited lexical competition for bilinguals but not monolinguals, we would need to demonstrate a three-way interaction: Tone * Word Type * Group. In error analysis, there was a main effect of Tone *(z = 3*.*10*, *p <* .*01*), suggesting that superimposed tones induced more overall difficulty in lexical processing. In addition, there was an interaction of Tone * Word Type (*z = 2*.*98*, *p <* .*01*), suggesting that superimposed tones induced more difficulty in processing IH words than non-IH words. However, there was no three-way interaction in error analysis, suggesting that the degree of difficulty in lexical processing for both groups was comparable across Experiment 1 and 2 in both IH and non-IH words *(z =* .*30*, *p =* .*76)*. In reaction time analysis, as demonstrated in Table 6, there was a main effect of Group *(t = 4*.*44*, *p<* .*001)*, a main effect of Tone *(t = 4*.*30*, *p <* .*001)*, as well as a main effect of Word Type *(t = 3*.*14*, *p <* .*01*). In addition, there are interactions of Group * Tone *(t = 2*.*04*, *p <* .*05)* and Tone * Word Type *(t = 3*.*40*, *p <* .*01)*, indicating that monolinguals and bilinguals behaved differently when perceiving superimposed tones and this tonal manipulation altered the responses to IH words compared to non-IH words. Importantly, we observed a three-way interaction of Group x Tone x Word Type *(t = 2*.*15*, *p =* .*035 <* .*05*), suggesting that lexical tones slowed down bilinguals’ responses to IHs, compared to non-IHs, but not much so for monolinguals. These results confirm our hypothesis that superimposed lexical tones guided bilingual lexical access to the non-target language, Mandarin, and caused slower responses to IHs as a consequence of cross-language lexical competition.

**Table 6:** Linear mixed-effects analysis results for both groups in Experiment 1 and 2.

Bilinguals and Monolinguals in Experiment 1 and 2						
*Random effects*:						
Groups	Name	Variance	Std.Dev.		Corr	
subject	(Intercept)	24727	157.25			
	wtypenon-IH	1432	37.85		-0.13	
itemN	(Intercept)	16665	129.09			
	pitchuntone	11730	108.30		-0.42	
	groupmono	6813	82.54		-0.25	
	pitchuntone:groupmono	5468	73.95		-0.12	
Residual		70906	266.28			
*Fixed effects*:						
	Estimate	Std. Error	Df	t value		Pr(>|t|)
(Intercept)	867.649	42.222	118.914	20.550		< 2e-16 ***
groupmono	-239.160	53.843	89.458	-4.442		2.54e-05 ***
pitchuntone	-230.781	53.636	95.360	-4.303		4.08e-05 ***
wtypenon-IH	-111.529	35.505	72.684	-3.141		0.00243 **
groupmono:pitchuntone	148.486	72.890	82.510	2.037		0.04484 *
groupmono:wtypenon-IH	2.827	32.570	62.966	0.087		0.93111
pitchuntone:wtypenon-IH	121.790	35.722	73.801	3.409		0.00106 **
groupmono:pitchuntone:wtypenon-IH	-84.217	39.213	66.522	-2.148		0.03539 *

.p < 0.1.

* p < 0.05.

** p < 0.01.

*** p < 0.001

Second, we ran linear mixed-effects analysis on *bilinguals* to understand whether the absence and presence of lexical tones on the same syllables would produce any significant change across Experiment 1 (without tones) versus 2 (with tones). The bilingual results showed a strong interaction between Word Type (IH vs. non-IH) and Tone in both error rate and reaction time analyses (*z = 2*.*79*, *p <* .*01* in error rates, *t = 3*.*09*, *p <* .*01* in reaction times), with a main effect of Tone in both error rate and reaction time analyses (*z = 3*.*16*, *p <* .*01*, *t = 3*.*85*, *p <* .*001*), as well as a main effect of Word Type in reaction time analysis only (*t = 2*.*67*, *p <* .*01)*, as in Table 7. In particular, this interaction indicated that the toned English words significantly slowed down their responses to the IH words compared to the non-IH words. These results suggested that the toned words elicited cross-language competition in lexical processing, confirming that the presence of lexical tones in Experiment 2 altered bilinguals’ responses to IHs compared to non-IHs.

**Table 7:** Linear mixed-effects analysis results for bilinguals in Experiment 1 and 2.

Bilinguals in Experiment 1 and 2						
Random effects:						
Groups	Name	Variance	Std.Dev.		Corr	
itemN	(Intercept)	20894	144.55			
	pitchuntone	13493	116.16		-0.50	
subject	(Intercept)	41533	203.80			
	wtypenon-IH	1776	42.15		0.35	
Residual		115104	339.27			
*Fixed effects*:						
	Estimate	Std. Error	Df	t value		Pr(>|t|)
(Intercept)	908.45	52.75	61.02	17.222		< 2e-16 ***
pitchuntone	-261.83	68.09	46.81	-3.845		0.000363 ***
wtypenon-IH	-108.84	40.81	65.97	-2.667		0.009617 **
pitchuntone:wtypenon-IH	127.53	41.18	53.11	3.097		0.003118 **

p < 0.1.

* p < 0.05.

** p < 0.01.

*** p < 0.001.

Finally, we ran linear mixed-effects analysis on *monolinguals* and the results showed a main effect of Word Type in both error and reaction time analyses *(z = 3*.*14*, *p <* .*01 and t = 2*.*95*, *p <* .*01*). However, there was neither main effect of Tone *(t = 1*.*61*, *p =* . *11)* nor interaction between Word Type and Tone *(t = 1*.*19*, *p =* .*24)* in reaction time analysis, as in Table 8. In error analysis, there was a main effect of Tone (*z = 4*.*92*, *p <* .*001*), as well as an interaction between Word Type and Tone *(z = 3*.*37*, *p <* .*001*). These monolingual results indicate that monolinguals’ responses to IHs vs. non-IHs remained the same regardless of whether the same words/items were presented with or without lexical tones.

**Table 8:** Linear Mixed-Effects analysis results for monolinguals in Experiment 1 and 2.

Monolinguals in Experiment 1 and 2						
Random effects:						
Groups	Name	Variance	Std.Dev.		Corr	
itemN	(Intercept)	14492.6	120.39			
	pitchuntone	11563.1	107.53		-0.70	
subject	(Intercept)	9920.1	99.60			
	wtypenon-IH	337.2	18.36		0.23	
Residual		35677.7	188.89			
*Fixed effects*:						
	Estimate	Std. Error	Df	t value		Pr(>|t|)
(Intercept)	587.11	31.37	81.47	18.718		<2e-16 ***
pitchuntone	-60.40	37.52	58.98	-1.610		0.1128
wtypenon-IH	-91.90	31.19	68.04	-2.946		0.0044 **
pitchuntone:wtypenon-IH 36.26	30.53	63.70	1.188	0.2393		

.p < 0.1.

* p < 0.05.

** p < 0.01.

*** p < 0.001.

## General discussion

This current study was designed to address the role of lexical tones in bilingual spoken word recognition. In Experiment 1, we manipulated the phonological overlap between Mandarin and English words to test whether Mandarin-English bilinguals were sensitive to segmental overlap so as to induce language co-activation when listening to English words only. The results showed no main effects of inter-lingual homophones (IH) for bilinguals, but a main effect of IH for monolinguals. If language co-activation occurred, we would expect IH inhibitory effects for bilinguals due to the cross-language lexical competition from L1-Mandarin. In addition, the delay observed in IHs in monolinguals could not be due to cross-language lexical competition, because the English monolinguals had no knowledge of Mandarin. Clearly, bilinguals and monolinguals treated the same stimuli differently, which suggests that bilinguals and monolinguals use different processing strategies when listening to the same items in English. We will return to this difference later. In Experiment 2, we superimposed lexical tones onto the same English words used in Experiment 1. It was predicted that if lexical access was language non-selective with the presence of lexical tones, the bilinguals would demonstrate IH inhibitory effects, as in previous studies [10]. In the light of the findings in Experiment 1, the monolinguals should produce a similar pattern, as lexical tones should not affect monolinguals. The results appeared to confirm the predictions. Namely, the IH inhibitory effects emerged in bilinguals, and the monolinguals continued to respond to the non-IHs more quickly. To summarize the results, we only observed lexical competition from the non-target language (L1 Mandarin) with the presence of lexical tones in Mandarin-English bilinguals when they recognized the target language (L2 English) in the auditory modality. These results suggest the critical role of lexical tones in guiding lexical access for a tonal language when bilinguals are processing a non-tonal language. In the case of Mandarin-English bilinguals, whether lexical access is language selective (e.g., as in Ex 1) or language non-selective (e.g., as in Ex 2) can be constrained by language-specific features (i.e., lexical tones).

How do we account for the delay on IHs observed in monolinguals in both experiments? Importantly, this pattern was consistent across Exp 1 and Exp 2 in monolinguals. In an ideal situation, we would expect null effects with the monolinguals to demonstrate a straightforward comparison to the bilinguals. In constructing the materials, there was an inherent constraint in matching the number of phonemes between the IHs and the non-IHs, due to the difference in syllabic structure between Mandarin and English. As discussed earlier, Mandarin syllables predominantly follow the CV (consonant-vowel) structure, with the only exceptions being nasals /n/ and /ŋ/ in the coda position [35]; while English syllables mainly follow the CVC (consonant-vowel-consonant) structure [58]. In other words, in the current experiments, we were unable to match the number of phonemes between the IHs and non-IHs, generating most IHs being CV structures and all non-IHs being CVC structures when matching frequency and phonological neighborhood density item-by-item (see details in Table 2). In spoken word recognition for native listeners of English, the coda (i.e., final consonant) plays a critical role in both TRACE [5] and Cohort [2] models, as there was a very small number of CV monosyllabic words in English. That is, most monosyllabic English words are closed syllables and native listeners are biased towards closed syllabic words rather than open syllabic words. In TRACE, the coda provides an additional cue to select a word candidate; while in the Cohort model, open syllables potentially generate larger cohorts so that selecting word candidates would take longer. Therefore, both models predict that monosyllabic words of CVC structure are easier and faster to process than words of CV structure for English native listeners. This is exactly what we observed in both experiments: native listeners of English responded to the non-IH items (CVC structure) more quickly than the IH (CV structure) words. It is important to note that the slower responses to IH items in monolinguals still provide a useful comparison across two groups as we now know that the absence of IH inhibitory effects in bilinguals in Exp 1 is not confounded by the nature of stimuli (i.e. the IH items generated slower responses for native listeners).

But why did the bilinguals fail to show a similar inhibitory effect on the IH items in Experiment 1, opposite to Experiment 2? The contrast demonstrated by the bilinguals and monolinguals in Experiment 1 suggests that bilinguals used a different processing strategy in L2, which might be influenced by their L1. In fact, it is well documented in the literature that L1 phonological structures could have a persistent impact on L2 processing skills [30] [59–65]. For example, Nguyen-Hoan and Taft [65] observed that even early bilinguals in Australia (mean age of arrival: 1.64–2.24 years) who became English-dominant showed L1 influence in their phonological processing in L2. In a phoneme deletion task (Exp.1), participants of various L1 background were asked to delete the first or final ‘sound’ of a monosyllabic word or non-word in an utterance (e.g. *flat* or *flaz*). It was observed that monolingual English speakers deleted the most phoneme-sized sounds, implying that their interpretation of a ‘sound’ was at the phonemic level. In contrast, L1-Chinese and L1-Vietnamese participants tended to delete larger units than phonemes, which suggests that these bilinguals’ phonological processing was affected by their morpho-syllabic L1. Thus, Experiment 1 showed that our English listeners were sensitive to coda, while Mandarin listeners were not. This difference is also consistent with the metrical segmentation strategy [66], which proposes that listeners exploit the lexical statistics of the language in speech segmentation. In this case, English listeners were sensitive to phonemes, while Mandarin listeners were sensitive to syllables in identifying word boundaries.

This analysis is crucial in understanding the IH inhibitory effects observed in bilinguals in Experiment 2, further confirming the inhibition was due to cross-language lexical competition induced by both lexical tones and segments. The logic is that if bilingual listeners were sensitive to coda or to segmental overlap between L1 Mandarin and L2 English, we would expect to observe inhibition on the IH items in Experiment 1. However, this was not what we observed here. The contrast demonstrated between Experiments 1 and 2 in bilinguals suggests that language-specific cues (i.e., lexical tones) are crucial in bilingual lexical access with bilinguals whose one language is tonal while the other is non-tonal. That is, both tonal and segmental information need to be available to induce language co-activation. These findings differ from those from previous studies of bilingual lexical access by showing that segmental overlap was insufficient to activate the non-target Mandarin lexical representations. This is in line with the results reported by Lee [37], who found that segmental overlap alone between a prime and target was not sufficient to generate priming in Mandarin spoken word recognition. Two reasons can be proposed for the inability of segmental overlap alone to activate the Mandarin lexical representations. First, lexical tones have a comparable role to segments in Mandarin spoken word recognition [38]. Thus, lexical activation in Mandarin requires both segmental and supra-segmental information. Second, a large number of within-language homophones in Mandarin may encourage more distribution of cue weights, not only to tones but also to other information such as the context or the adjacent syllables in order to allow efficient recognition [36].

In addition, at the theoretical level, our current findings shed light on the mechanism of pitch processing. A large but under-researched theoretical debate in the literature of Speech Perception is whether the processing of pitch contours during word recognition is language context sensitive [67,68]. Here, language context could be the language mode of communication (e.g., the language of the conversation) or some specific acoustic-phonetic cues within a word or sentence. In the case of Mandarin-English bilinguals, lexical tones are an important cue to a word’s language membership, in particular, during code-switching. The current study was conducted entirely in an English context, with the instructions being given in English, as well as directing participants to treat the stimuli as English. However, this extra-word language context did not restrict the lexical processor from accessing the Mandarin lexicon provided with language-specific phonetic cues (i.e., lexical tones in Ex. 2). In other words, the within-word tonal information overrode the extra-word language context and guided lexical access to the non-target language. These results are in line with the findings of Quam and Creel (2017) with adult Mandarin-English bilinguals showing that within-word phonetic cues were more consequential than extra-word language context for language-specific phonological encoding, like lexical tones. This conclusion is also confirmed with findings from child bilinguals reported by Singh and Quam [68], showing children of 4–5 years old capable of integrating lexical tones into word meanings given within-word cues in addition to extra-word language context, but not extra-word language context alone.

One limitation of the current study is the unnaturalness of Experiment 2, as English stimuli were superimposed with lexical tones. As demonstrated earlier, the error data indicate that Experiment 2 turned out to be more difficult than Experiment 1 overall and IH words became more difficult than non-IH words with the superimposed pitch contours; in other words, the Mandarin pitch contours carried by English monosyllables appeared to ‘interfere’ with lexical processing in general terms for both monolinguals and bilinguals. Our post-experiment debriefing found that some participants reported difficulty in recognizing some items in Experiment 2. One way to improve this study methodologically is to manipulate pitch contour in the way that is consistent with English input in natural speech. For instance, English words can be pronounced with a pitch contour that is similar/comparable to Tone 2 or Tone 4 in Mandarin, whereas; Tone 3 is rarely observed in English pitch contour. Thus, our stimuli superimposed with Tone 3 can bias bilinguals more towards the Mandarin lexicon and generate more lexical competition. On the other hand, they could be a lot more difficult for monolinguals to process due to their unnaturalness in English speech.

The current study adds to the bilingual literature by providing empirical evidence of a linguistic dimension (i.e., supra-segmental information) as an important representational and processing mechanism in bilingual spoken word recognition. The contrast between Experiments 1 and 2 shows that lexical tones are a critical cue inducing cross-language lexical competition, in addition to segmental overlap across languages, as demonstrated in previous studies [10], [33]. It is worth noting that this result is consistent and complementary to recent work by Wang et al. [45], where lexical tones are mandatory in eliciting cross-language lexical competition during unconscious translation even when the English input contains no overlap with the non-target language Mandarin. Thus, with or without overt phonological overlap between the target and non-target language, for tonal bilinguals, supra-segmental information is crucial in activating lexical representation in the non-target tonal language. With regard to modeling bilingual spoken word recognition, the current data add support for the modified TRACE-T model [69], which encodes Mandarin tones and phonemes. According to the TRACE-T model, among the three layers of representation, the middle one consists of both phonemes and tones, and continuous mapping takes place to the relevant representations as listeners receive input. To extend this model to the bilingual situation, cross-language activation requires bottom-up acoustic-phonetic information that overlaps cross-linguistically at both segmental and supra-segmental levels (i.e., interlingual homophones), which could in turn be mapped to specific Mandarin representations at the word/lexical level. These activated representations then induce cross-language competition at the lexical level when they laterally inhibit irrelevant candidates in both languages, causing delay compared to non-IHs whose inhibition only involves one language.

## Conclusion

In summary, our findings support the requirement of a precise input-representation match for language co-activation and hence cross-linguistic interactions, echoing Ju and Luce [9] in the auditory modality. In addition, this precise match should be considered at multiple levels, from the sub-phonemic to the supra-segmental. That is, language-specific phonetic features may affect cross-language activation. Thus, the mechanism of bilingual lexical access can depend on these features in terms of language selectivity or non-selectivity in the auditory domain. Our results contribute to the literature in two ways. First, we demonstrate that L1-Mandarin—L2 English bilinguals only showed lexical competition from the non-target language when both tonal and segmental information were available in the target language, thus suggesting language selective or non-selective access can be constrained by language-specific cues. Second, we demonstrate that the difference between Mandarin-English bilinguals and English monolinguals in their phonological processing strategy is due to L1 transfer, when L1 Mandarin is syllable-based and L2 English is phoneme-based.

## Supporting information

S1 Data (ZIP)

S1 Appendix Identification of mandarin inter-lingual homophones with corresponding English words (Chinese characters). (DOCX)

S2 Appendix Interlingual homophones (IH) selected with similarity ratings. (DOCX)

S3 Appendix The list of IHs (inter-lingual homophones) and non-IHs. (DOCX)
