# Supplementary material for: Language selective or non-selective in bilingual lexical access? It depends on lexical tones!
Source: PLoS One. 2020 Mar 23;15(3):e0230412. doi: 10.1371/journal.pone.0230412 (PMC7089543; doi:10.1371/journal.pone.0230412)
Supplement: S1 Appendix — (DOCX) [file pone.0230412.s002.docx]

# Appendix A: Identification of Mandarin inter-lingual homophones with corresponding English words (Chinese characters)

|  | **/a/ or /a:/** | **/aɪ̯/ or /ʌɪ/** | **/an/ or /a:n/** | **/ɑʊ̯/ or /ɑu/** | **/eɪ̯/ or /ei/** | **/i:/ or /i/** | **/in/ or /i:n/** | **/u/ or /u:/** | **/ ɔ / or /ɔ:/ or /o/** | **/oʊ/** |
| --- | --- | --- | --- | --- | --- | --- | --- | --- | --- | --- |
| **/b/ or /p/** | bar (把) | buy (白) | barn (班) | bow (包) | bay (杯) | bee (逼) | bin (宾) |  |  |  |
| **/p/ or /pʰ/** | par (怕) | pie (拍) |  |  | pay (陪) |  | pin (拼) | poo (扑) |  |  |
| **/m/** |  | my (买) |  |  | May (没) | me (米) | mean (民) |  |  |  |
| **/f/** | far (发) |  |  |  |  |  |  |  |  |  |
| **/d/ or /t/** |  | die (待) | darn (单) |  | day (得) |  |  | do (都) |  | dough (豆) |
| **/t/ or /tʰ/** | tar (他) | tie (胎) |  |  |  | tea (梯) |  | two (秃) |  |  |
| **/l/** |  | lie (赖) |  |  | lay (累) | Lee (里) | lean (林) | loo (路) |  | low(漏) |
| **/n/** |  |  |  | now (脑) |  |  |  |  |  | know (耨) |
| **/g/ or /k/** |  | guy (盖) |  |  | gay (给) |  |  |  |  | go (沟) |
| **/k/ or /kʰ/** | car (卡) |  |  | cow (靠) |  |  |  |  |  |  |
| **/x/ or /h/** |  | high (海) |  | how (好) | hey (黑) |  |  | who (湖) |  |  |
| **/s/** |  |  |  |  |  |  |  | sue (苏) |  | so (搜) |
| **/r/ or /ɹ/** |  |  |  | row (饶) |  |  |  |  |  | row (柔) |
| **/w/** |  | why (歪) |  |  | way (位) |  |  |  | war (窝) |  |
