# Supplementary material for: Language selective or non-selective in bilingual lexical access? It depends on lexical tones!
Source: PLoS One. 2020 Mar 23;15(3):e0230412. doi: 10.1371/journal.pone.0230412 (PMC7089543; doi:10.1371/journal.pone.0230412)
Supplement: S2 Appendix — (DOCX) [file pone.0230412.s003.docx]

# Appendix B. Interlingual homophones (IH) selected with similarity ratings

| English targets | IPA of English targets | Mandarin Character of the IHs | Pinyin of the IHs | IPA of the IH in Mandarin | Similarity Rating (Means) |
| --- | --- | --- | --- | --- | --- |
| me | /miː/ | 密 | mi4 | /mi4/ | 5.4 |
| my | [/mʌɪ/](http://oxforddictionaries.com/words/key-to-pronunciation) | 买 | mai3 | /maɪ̯3/ | 4.8 |
| do | [/duː/](http://oxforddictionaries.com/words/key-to-pronunciation) | 读 | dou4 | /tɤʊ̯4/ | 4.3 |
| so | [/səʊ/](http://oxforddictionaries.com/words/key-to-pronunciation) | 搜 | sou1 | /sɤʊ̯1/ | 5.6 |
| go | [/gəʊ/](http://oxforddictionaries.com/words/key-to-pronunciation) | 够 | gou4 | /kɤʊ̯4/ | 5.8 |
| now | [/naʊ/](http://oxforddictionaries.com/words/key-to-pronunciation) | 脑 | nao3 | /nɑʊ̯3/ | 5.1 |
| how | [/haʊ/](http://oxforddictionaries.com/words/key-to-pronunciation) | 好 | hao4 | /xɑʊ̯4/ | 5.3 |
| why | [/wʌɪ/](http://oxforddictionaries.com/words/key-to-pronunciation) | 外 | wai4 | /wu̯aɪ̯4/ | 5.0 |
| who | /huː/ | 护 | hu4 | /xu4/ | 5.7 |
| way | [/weɪ/](http://oxforddictionaries.com/words/key-to-pronunciation) | 位 | wei4 | /wu̯e4ɪ̯/ | 5.0 |
| mean | [/miːn/](http://oxforddictionaries.com/words/key-to-pronunciation) | 民 | min3 | /min3/ | 4.6 |
| two | [/tuː/](http://oxforddictionaries.com/words/key-to-pronunciation) | 图 | tu2 | /tʰu2/ | 5.2 |
| guy | [/gʌɪ/](http://oxforddictionaries.com/words/key-to-pronunciation) | 该 | gai1 | /kaɪ̯1/ | 5.6 |
| car | [/kɑː/](http://oxforddictionaries.com/words/key-to-pronunciation) | 卡 | ka3 | /kʰɑ3/ | 4.0 |
| die | [/dʌɪ/](http://oxforddictionaries.com/words/key-to-pronunciation) | 带 | dai4 | /taɪ̯4/ | 4.4 |
| high | [/hʌɪ/](http://oxforddictionaries.com/words/key-to-pronunciation) | 还 | hai2 | /xaɪ̯2/ | 4.0 |
| buy | [/bʌɪ/](http://oxforddictionaries.com/words/key-to-pronunciation) | 白 | bai2 | /paɪ̯2/ | 5.3 |
| lie | [/lʌɪ/](http://oxforddictionaries.com/words/key-to-pronunciation) | 来 | lai2 | laɪ̯2/ | 6.4 |
| bar | [/bɑː/](http://oxforddictionaries.com/words/key-to-pronunciation) | 把 | ba4 | /pɑ4/ | 4.0 |
| low | [/ləʊ/](http://oxforddictionaries.com/words/key-to-pronunciation) | 楼 | lou2 | /lɤʊ̯2/ | 5.1 |
| gay | [/geɪ/](http://oxforddictionaries.com/words/key-to-pronunciation) | 给 | gei3 | keɪ̯3/ | 4.8 |
| tea | [/tiː/](http://oxforddictionaries.com/words/key-to-pronunciation) | 题 | ti2 | /tʰi2/ | 4.8 |
| lay | [/leɪ/](http://oxforddictionaries.com/words/key-to-pronunciation) | 类 | lei4 | /leɪ̯4/ | 5.0 |
| tie | [/tʌɪ/](http://oxforddictionaries.com/words/key-to-pronunciation) | 台 | tai2 | /tʰaɪ̯2/ | 5.4 |
| sue | [/suː/](http://oxforddictionaries.com/words/key-to-pronunciation) | 速 | su4 | /su4/ | 5.2 |
| pie | [/pʌɪ/](http://oxforddictionaries.com/words/key-to-pronunciation) | 排 | pai2 | /pʰaɪ̯2/ | 5.5 |
| row | [/rəʊ/](http://oxforddictionaries.com/words/key-to-pronunciation) | 肉 | rou4 | /ʐɤʊ̯4/ | 4.7 |
| cow | [/kaʊ/](http://oxforddictionaries.com/words/key-to-pronunciation) | 考 | kao3 | /kʰɑʊ̯3/ | 5.3 |
| bay | [/beɪ/](http://oxforddictionaries.com/words/key-to-pronunciation) | 被 | bei4 | /peɪ̯4/ | 5.3 |
| pin | [/pɪn/](http://oxforddictionaries.com/words/key-to-pronunciation) | 品 | pin3 | /pʰin3/ | 4.6 |
| dough | [/dəʊ/](http://oxforddictionaries.com/words/key-to-pronunciation) | 都 | dou1 | /tɤʊ̯1/ | 4.6 |
| toe | [/təʊ/](http://oxforddictionaries.com/words/key-to-pronunciation) | 头 | tou2 | /tʰɤʊ̯2/ | 5.0 |
| lean | [/liːn/](http://oxforddictionaries.com/words/key-to-pronunciation) | 林 | lin2 | /lin2/ | 5.4 |
| bee | [/biː/](http://oxforddictionaries.com/words/key-to-pronunciation) | 必 | bi4 | /pi4/ | 4.8 |
| bin | [/bɪn/](http://oxforddictionaries.com/words/key-to-pronunciation) | 宾 | bin1 | /pin1/ | 5.7 |
| par | [/pɑː/](http://oxforddictionaries.com/words/key-to-pronunciation) | 怕 | pa4 | /pʰɑ4/ | 4.3 |
| tar | [/tɑː/](http://oxforddictionaries.com/words/key-to-pronunciation) | 他 | ta1 | /tʰɑ1/ | 4.1 |
