# Supplementary material for: Language selective or non-selective in bilingual lexical access? It depends on lexical tones!
Source: PLoS One. 2020 Mar 23;15(3):e0230412. doi: 10.1371/journal.pone.0230412 (PMC7089543; doi:10.1371/journal.pone.0230412)
Supplement: S3 Appendix — (DOCX) [file pone.0230412.s004.docx]

# Appendix C The list of IHs (inter-lingual homophones) and non-IHs

| **No.** | **IH** | **No.** | **Fillers (Non-IH)** |
| --- | --- | --- | --- |
| 1 | me | 38 | what |
| 2 | my | 39 | your |
| 3 | do | 40 | have |
| 4 | so | 41 | but |
| 5 | go | 42 | like |
| 6 | now | 43 | come |
| 7 | how | 44 | one |
| 8 | why | 45 | good |
| 9 | who | 46 | back |
| 10 | way | 47 | make |
| 11 | mean | 48 | need |
| 12 | two | 49 | thing |
| 13 | guy | 50 | call |
| 14 | car | 51 | hell |
| 15 | die | 52 | hit |
| 16 | high | 53 | line |
| 17 | buy | 54 | hot |
| 18 | lie | 55 | win |
| 19 | bar | 56 | rock |
| 20 | low | 57 | cat |
| 21 | gay | 58 | sad |
| 22 | tea | 59 | lock |
| 23 | lay | 60 | raise |
| 24 | tie | 61 | rain |
| 25 | sue | 62 | laid |
| 26 | pie | 63 | wise |
| 27 | row | 64 | rise |
| 28 | cow | 65 | sale |
| 29 | bay | 66 | rate |
| 30 | pin | 67 | mass |
| 31 | dough | 68 | goal |
| 32 | toe | 69 | sack |
| 33 | lean | 70 | lip |
| 34 | bee | 71 | lick |
| 35 | bin | 72 | mock |
| 36 | par | 73 | sip |
| 37 | tar | 74 | hen |
